# Nuclear STAT3 expression is associated with favorable prognosis in papillary thyroid carcinoma

**DOI:** 10.1530/ETJ-25-0080

**Published:** 2025-11-03

**Authors:** Chie Masaki, Tomohiro Chiba, Satoko Baba, Kazuma Moriya, Aya Ebina, Kazuhisa Toda, Hiroki Mitani, Tomoo Jikuzono, Ryuji Ohashi, Kiminori Sugino, Koichi Ito, Iwao Sugitani, Kengo Takeuchi

**Affiliations:** ^1^ Department of Pathology, Cancer Institute Hospital, Japanese Foundation for Cancer Research, Tokyo, Japan; ^2^ Department of Endocrine Surgery, Nippon Medical School Hospital, Tokyo, Japan; ^3^ Department of Surgery, Ito Hospital, Tokyo, Japan; ^4^ Department of Cytology, Cancer Institute Hospital of Japanese Foundation for Cancer Research, Tokyo, Japan; ^5^ Ariake Minna Clinic, Tokyo, Japan; ^6^ Division of Head and Neck, Cancer Institute Hospital of Japanese Foundation for Cancer Research, Tokyo, Japan; ^7^ Department of Integrated Diagnostic Pathology, Nippon Medical School, Tokyo, Japan

**Keywords:** signal transducer and activator of transcription 3 (STAT3), papillary thyroid carcinoma (PTC), prognosis, immunohistochemistry (IHC), tissue microarray (TMA)

## Abstract

**Introduction:**

Signal transducer and activator of transcription 3 (STAT3) is a signaling molecule that functions downstream of various cytokine and growth factor receptor signaling pathways to regulate cell growth, survival, and differentiation. Constitutive activation of STAT3 is relevant to cancer development and progression in many types of malignancies. In this study, the relationship between STAT3 activation and the prognosis of papillary thyroid carcinoma (PTC) was retrospectively examined using immunohistochemical staining with an anti-STAT3 antibody.

**Materials and methods:**

A total of 1,132 PTC cases with M0 diagnosed between 1993 and 2012 were included. The H-score (0–300 points) was used to evaluate nuclear staining of STAT3 (n-STAT3), which reflects the activated form of STAT3. The relationship between the n-STAT3 score and recurrence-free survival (RFS) was examined.

**Results:**

The median n-STAT3 score was 105. RFS was compared between the two groups using the Kaplan–Meier method at a cutoff of 70 points (AUC = 0.60), calculated from the ROC curve based on recurrence. Ten-year and 20-year RFS were 87.5 and 83.1% in the high n-STAT3 group (*n* = 764) and 77.7 and 72.6% in the low n-STAT3 group (*n* = 371), respectively. The high n-STAT3 group had a significantly better RFS (*P* < 0.0001). Age (≥55 years), tumor size (≥4 cm), extrathyroidal extension, larger maximum lymph node size (≥30 mm), and low n-STAT3 levels were associated with poor RFS on univariate and multivariate analyses.

**Conclusion:**

n-STAT3 scores in cases with PTC were associated with favorable prognosis. Further studies are required to elucidate the mechanism.

## Introduction

The prognosis of papillary thyroid carcinoma (PTC) is wide-ranging. Risk stratification and ‘risk-adapted management’ (1), such as watchful waiting for low-risk papillary thyroid microcarcinoma (PTMC) based on its good life expectancy, and molecular-targeted therapies for high-risk groups, are representative treatment strategies in recent years (2, 3). Although PTC frequently involves lymph node metastasis (LNM), it is not necessarily fatal. Only a limited number of cases, even those with distant metastasis, have fatal potential in a short time, and most require long-term follow-up. Furthermore, even cases without distant metastases at diagnosis can have a fatal course, since the metastases extend over the cervical LNs, grow, and progress to distant metastases. Therefore, biomarkers that can predict disease progression or mortality at the time of diagnosis would be ideal. Recent developments in molecular markers are expected to identify markers associated with recurrence and progression, such as *BRAF* p.V600E and *TERT* promoter mutations (4). In this study, we focused on members of the STAT family, which we believe to be promising candidates for such markers.

STAT3 is a key effector of cytokine receptor/JAK/STAT signaling and is phosphorylated on Tyr705 by kinases such as JAK2 in response to ligand–receptor interactions. Phosphorylated STAT3 (pSTAT3) enters the nucleus and binds to DNA sequences called gamma-activated sequences as a transcription factor, inducing the expression of genes involved in the development, progression, and/or immune evasion of cancer (2, 3). Genetic/epigenetic alterations underlie aberrant STAT3 signaling in cancer cells. Many types of malignancies have been found to show STAT3 hyperactivation, and high levels of pSTAT3 have been reported to correlate with poor clinical outcomes (5, 6, 7). However, there are conflicting results for the clinical impact of STAT3 on thyroid cancer prognosis; some studies showed a poor prognosis, similar to previous reports (8), whereas others showed a favorable prognosis (9, 10).

In this study, we evaluated nuclear STAT3 (n-STAT3), a marker of activated STAT3, in PTC cells to investigate the prognostic significance of STAT3 activation.

## Materials and methods

### Data selection

PTC cases that were surgically resected and pathologically diagnosed between 1993 and 2012 at Cancer Institute Hospital of Japanese Foundation for Cancer Research, except for papillary thyroid microcarcinoma (maximal diameter ≤1.0 cm), were included in this single-institutional study. Cases with any distant metastasis at diagnosis (M1) were excluded because recurrence-free survival (RFS) was used as a prognostic outcome indicator in this analysis. Tumor recurrence was defined as new evidence of locoregional disease or distant metastases occurring more than 6 months after successful primary therapy. Tissue microarray (TMA) analysis was performed by selected board-certified pathologists for each thyroid primary lesion and LNM, in which LNs were sampled only if there was more than one metastasis. The selected points of the donor paraffin blocks were punched with a 1 mm-diameter coring needle and transferred to the array in the recipient block using a manual tissue arrayer (KIN-1; Azumaya, Japan). Tissue sections with thickness of 4 μm were used for immunohistochemistry. Arrays with inadequate STAT3 staining due to decalcification using an acidic solution and those with fewer than 200 cancer cells per array were excluded.

### Immunohistochemistry

STAT3 is typically located in the cytoplasm and is promptly translocated to the nucleus on activation. Since the STAT3 antibody stains both non-phosphorylated STAT3 and phosphorylated STAT3 (pSTAT3), STAT3 in the nucleus (n-STAT3) is considered an active form of STAT3 (11, 12).

The expression of STAT3 and pSTAT3(Y705) was evaluated by immunohistochemistry (IHC) using TMAs. Following deparaffinization, the sections were pretreated at 95 °C for 30 min in Tris–EDTA buffer solution (pH 9.0) for antigen retrieval. Endogenous peroxidase activity was blocked using 3% hydrogen peroxide solution (FUJIFILM Wako Pure Chemical Industries, Ltd., Japan) for 5 min at room temperature. The sections were then incubated with an anti-STAT3 mouse monoclonal antibody (124H6, Cell Signaling Technology (USA) at a 1:300 dilution) and a pSTAT3(Y705) rabbit monoclonal antibody (D3A7; Cell Signaling Technology (1:100 dilution) for 30 min at room temperature using a Nichirei Histostainer (Nichirei Biosciences Inc., Japan). Immunoreactivity was visualized using Histofine Simple Stain MAX PO (mouse; Nichirei Biosciences, Inc.) with 3,3′-diaminobenzidine (DAB). For the anti-p-STAT3(Y705) antibody, we also used a modified peroxidase-antiperoxidase method to increase sensitivity (13). The sections were then counterstained with hematoxylin at room temperature for 1 min. The intensity and ratio of n-STAT3-stained carcinoma cells, not in immune cells or mesenchymal cells, including endothelial cells and fibroblasts, were scored using the H-score, a semi-quantitative method (Supplementary Fig. 1 (see section on Supplementary materials given at the end of the article)). The H-score was determined as the sum of (each category of immunoreactive intensity from 0 to 3) × (percentage of tumor cells in each category) (for example, 1 × 50% + 2 × 50% = 150). The results ranged from 0 to 300, where 0 indicated that all cells were negative, and 300 indicated that all cells were strongly positive. Each array was visually scored by two independent observers (C M and T C).

To confirm reliability and reproducibility, we performed STAT3 immunohistochemistry in two human PTC cell lines (TPC-1 and WRO) with or without IL-6 treatment and found that IL-6 treatment efficiently induced n-STAT3 in IL-6-treated cells (Supplementary Fig. 2). In addition, analysis of 12 whole-slide specimens demonstrated that n-STAT3 expression showed minimal intratumoral heterogeneity in PTC cells (Supplementary Fig. 3).

### Receiver-operating characteristic (ROC) curve analysis of n-STAT3 scores

The H-score distribution of the two groups according to recurrence was analyzed. The cutoff value of the n-STAT3 score for recurrence was investigated using a ROC curve. The cases were divided into two groups according to the n-STAT3 score. The impact of clinicopathological features on the two groups, according to the n-STAT3 score, was also investigated. The effects of each clinical factor and the n-STAT3 score on RFS were analyzed using univariate and multivariate logistic regression analyses.

### Relationship between n-STAT3 and genetic factors

Of the 1,132 cases, *BRAF* p.V600E point mutation and *TERT* promoter mutation by direct sequencing had already been carried out only for cases in which surgery was performed after January 2001 (546 cases) (4). The relationships of *BRAF* p.V600E point mutation and *TERT* promoter mutation with the n-STAT3 score were investigated.

### Statistical analysis

Data were analyzed using statistical software (JMP software v17.0, SAS Institute, Inc., USA). Categorical variables were compared using Fisher’s exact test, and continuous variables were compared using Welch *t*-test. Multivariate analysis was performed using all variables regardless of *P*-value on univariate analysis. Between-group differences in patient characteristics were tested for significance. An RFS curve was constructed using the Kaplan–Meier method. Significance was set at *P* < 0.05.

## Results

### Recruitment of cases

**Figure 1:**
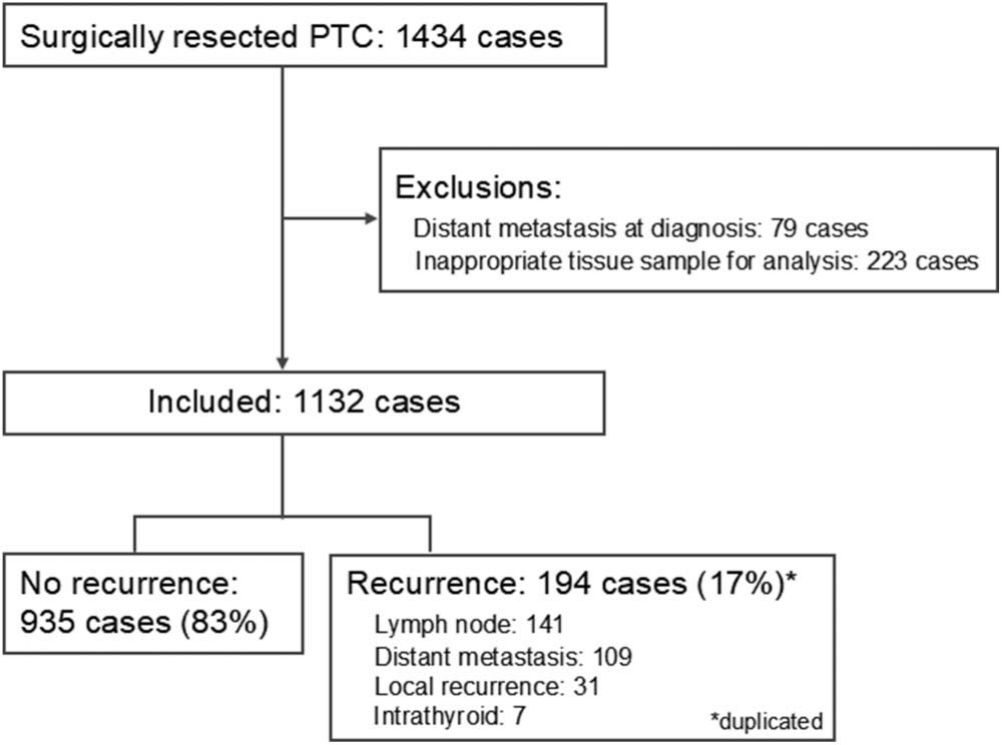
Recruitment of cases. Of the PTC cases that were surgically resected and diagnosed, 1,132 were evaluated after the exclusion of cases with any distant metastasis at diagnosis (M1) and cases that could not withstand STAT3 evaluation in this study.

Of the 1,434 surgically resected PTC cases, 179 cases of M1 at diagnosis and 223 cases that could not withstand STAT3 evaluation were excluded. A total of 1,132 cases were confirmed in the study (Fig. 1). Patient demographics and tumor characteristics are shown in the left-most column of Table 1. The mean age was 53.3 ± 14.3 years, the proportion of females was 78% (884), and the mean observation period (range) after the initial surgery was 13.5 (0.4–29.2) years. Of them, 194 cases (17%) had any recurrence. LNM was the most frequent type of recurrence (141 cases), followed by distant metastasis (109 cases), of which 42 (3.7%) had distant metastasis alone. Disease-specific death occurred in 52 (4.6%) cases, of which 40 died of distant metastases, and 12 died of local recurrence alone.

**Table 1:** Correlations between clinicopathological features and n-STAT3 expression. Bold values indicate statistical significance at *P<*0.05.

	All	n-STAT3 expression		*P*-value
		High	Low	
	*n* = 1,132	*n* = 763	*n* = 369	
Age, years	53.3 ± 14.3	53.5 ± 13.9	53.0 ± 15.2	0.5303
Age, ≥55 years	555 (49%)	386 (51%)	169 (46%)	0.1307
Sex				
Male	248 (22%)	165 (22%)	83 (22%)	0.7407
Female	884 (78%)	598 (78%)	286 (78%)	
Tumor size, mm	24.5 ± 14.6	23.2 ± 14.5	27.0 ± 14.5	**<0.0001**
Tumor size				
≥40 mm	148 (13%)	86 (11%)	62 (17%)	**0.0097**
<40 mm	974 (86%)	677 (89%)	307 (83%)	
Gross extrathyroidal extension				
Present	223 (20%)	142 (19%)	81 (22%)	0.1883
Absent	909 (80%)	621 (81%)	288 (78%)	
Clinical LNM				
N0 or N1a	788 (70%)	550 (72%)	238 (65%)	**0.0093**
N1b	344 (30%)	213 (27%)	131 (35%)	
Maximum size of LNM, mm	26.0 ± 14.2	25.3 ± 14.1	27.1 ± 14.5	0.1237
Maximum size of LNM				
≥3 cm	112 (10%)	67 (28%)	45 (34%)	0.2730
<3 cm	260 (23%)	171 (72%)	89 (66%)	
Number of LNMs	4.1 ± 5.8	3.6 ± 4.9	5.0 ± 7.2	0.0726
Number of LNMs				
≥5	339 (30%)	209 (27%)	130 (35%)	**0.0070**
<5	793 (70%)	554 (73%)	239 (65%)	
Recurrence				
Yes	195 (17%)	107 (14%)	88 (24%)	**<0.0001**
No	937 (83%)	656 (86%)	281 (76%)	

LNM, lymph node metastasis.

### n-STAT3 staining intensity

**Figure 2:**
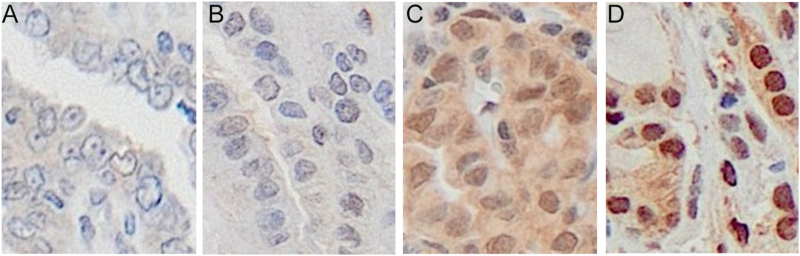
Representative images of n-STAT3 staining intensity by H-score. Staining intensity for each score was calculated as follows: (A) Score 0: the nuclei are completely unstained regardless of cytoplasmic staining. (B) Score 1: weakly positive nuclei. (C) Score 2: the staining intensity of each nucleus is intermediate between scores 1 and 3. (D) Score 3: nuclei are strongly positive.

We evaluated n-STAT3 staining in PTC cells, morphologically excluding n-STAT3 positivity in immune cells, endothelial cells, and fibroblasts (Supplementary Fig. 1). A representative image of n-STAT3 staining in primary PTCs, evaluated using the H-score, is shown in Fig. 2. The median n-STAT3 score for all 1,132 cases was 105 (0–280) points. An immunoreactive intensity category of ‘3’ was found in 525 cases (47%), including a small percentage of tumor cells. The highest intensity category was ‘2’ in 274 cases (24%) and ‘1’ in 331 cases (29%).

Immunohistochemistry using pSTAT3(Y705) was also performed, but only dim or inadequate staining intensity compared to that of n-STAT3 was obtained (Supplementary Fig. 4). Therefore, investigations using only n-STAT3 were conducted as a surrogate marker of activated STAT3.

### Distribution of n-STAT3 scores according to recurrence

**Figure 3:**
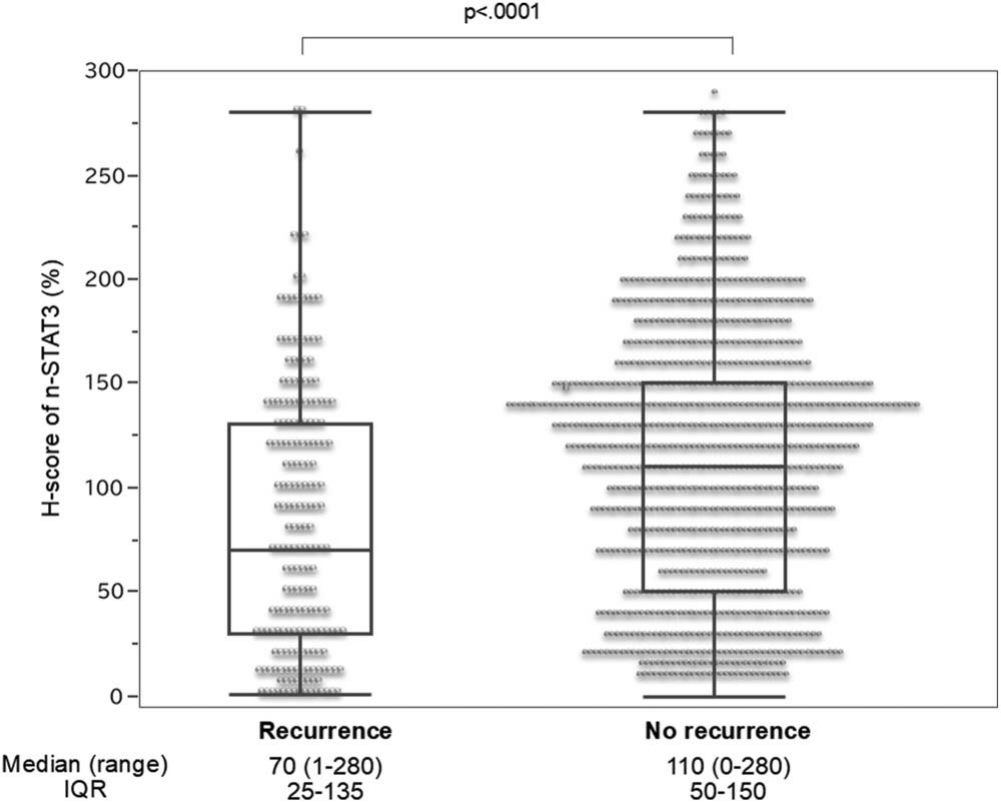
H-score distribution of n-STAT3 by recurrence. The median staining intensity scores of cases with and without recurrence are 110 (935 (83%) cases) and 70 (194 (17%) cases), respectively, indicating a significantly lower n-STAT3 score in the recurrence group.

The n-STAT3 scores were compared between the groups with and without recurrence (Fig. 3). The median n-STAT3 score in 935 (83%) cases without recurrence was 110 (range: 0–280, interquartile range (IQR): 50–150). However, it was 70 (range: 1–280, IQR: 25–135) in 194 (17%) cases with recurrence, indicating significantly lower n-STAT3 scores in the recurrence group (*P* < 0.0001). Next, based on the relationship between n-STAT3 scores and recurrence, a cutoff value of 70 for n-STAT3 scores (AUC = 0.598) was established using the ROC curve (Supplementary Fig. 5). Table 1 presents the results of the two-group comparison of each clinical factor between the high n-STAT3 group (≥70) and low n-STAT3 group (<70). The high n-STAT3 group had significantly smaller primary tumor sizes, a lower proportion of clinical N1b cases, and fewer LNMs (<5 nodes).

### Recurrence-free survival according to n-STAT3 score

**Figure 4:**
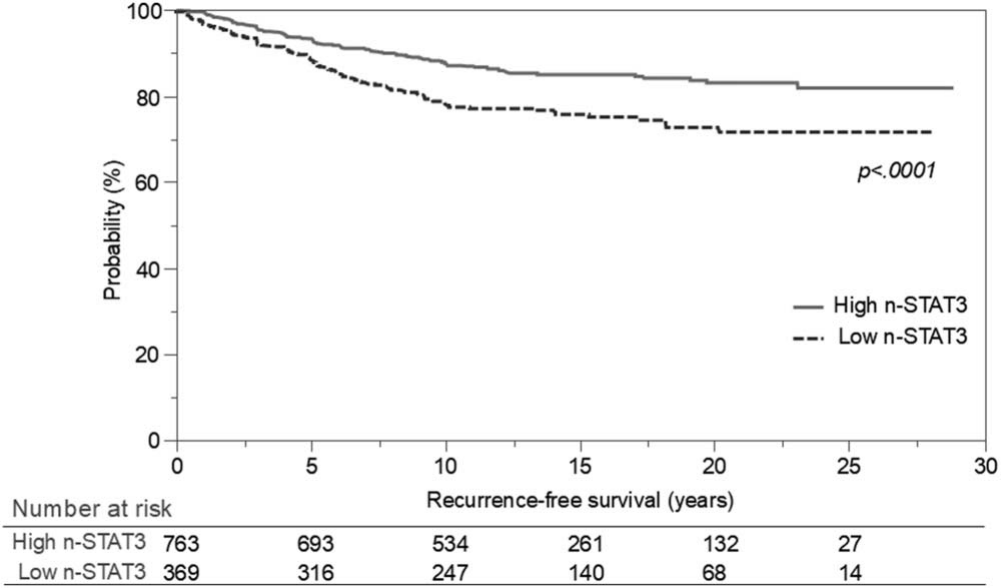
RFS according to n-STAT3 expression. The high n-STAT3 group shows significantly higher RFS (*P* < 0.0001). The 10-year and 20-year RFS rates are 87.5 and 83.1% for the high n-STAT3 group and 77.7 and 72.6% for the low n-STAT3 group, respectively.

Figure 4 shows a comparison of RFS rates by n-STAT3 score. The high n-STAT3 group showed significantly higher RFS (*P* < 0.0001). The 10-year and 20-year RFS rates were 87.5 and 83.1% for the high n-STAT3 group and 77.7 and 72.6% for the low n-STAT3 group, respectively.

### Correlations between clinicopathological features and recurrence-free survival

The relationships of representative clinical factors, n-STAT3 scores, and RFS were analyzed using univariate and multivariate analyses (Table 2). Clinical factors included age (≥55 years), sex (female), tumor size (≥4 cm), gross extrathyroidal extension (gross Ex, present), clinical N1b (present), maximum size of LNMs (≥30 mm), and number of LNMs (≥5). Univariate analysis showed significant differences in n-STAT3 scores (<70) along with all clinical factors, which were the same as those in previous reports from our institution. Of these, age (≥55 years), tumor size (≥4 cm), gross extrathyroidal extension (gross Ex, present), maximum LNM size (≥30 mm), and n-STAT3 score (<70) showed significant differences in the multivariate analysis.

**Table 2:** Univariate and multivariate logistic regression analyses for RFS. Bold values indicate statistical significance at *P<*0.05.

Factor	Univariate			Multivariate		
	HR	95% CI	*P*-value	HR	95% CI	*P*-value
Age, ≥55 years	3.110	2.292–4.279	**<0.0001**	3.976	2.620–6.148	**<0.0001**
Sex, male	1.699	1.240–2.299	**0.0007**	1.0714	0.719–1.578	0.7303
Tumor size, ≥4 cm	3.352	2.438–4.548	**<0.0001**	2.128	1.392–3.1953	**0.0004**
Ex, present	3.927	2.818–5.595	**<0.0001**	2.600	1.494–4.8616	**0.0014**
N1b, present	3.004	2.266–3.989	**<0.0001**	1.532	0.827–3.115	0.2030
Maximum size of LNMs, >30 mm	2.378	1.629–3.459	**<0.0001**	1.763	1.1878–2.616	**0.0048**
Number of LNMs, ≥5	2.331	1.757–3.090	**<0.0001**	1.202	0.763–1.948	0.4400
H-score of STAT3, ≥70	0.559	0.422–0.743	**<0.0001**	0.659	0.4510–0.968	**0.0320**

CI, confidence interval; HR, hazard ratio; LNM, lymph node metastasis; RFS, recurrence-free survival.

### Relationships of *BRAF* p.V600E and *TERT* promoter mutations with n-STAT3

Of 546 (48%) cases with *BRAF* p.V600E and *TERT* promoter mutations that had already been investigated, *BRAF* p.V600E was found in 442 (81%) cases, and *TERT* promoter mutations were found in 94 (17%) cases. The high n-STAT3 cases were significantly more frequent in *BRAF* p.V600E-positive 318 (72%) cases than in negative 60 (58%) cases (*P* = 0.0039), and in *TERT* promoter-negative 320 (71%) cases than in positive 56 (60%) cases (*P* = 0.0229) (Supplementary Fig. 6).

## Discussion

The relationship between the n-STAT3 scores in PTC and prognostic outcomes using RFS was investigated in this study. The results showed that higher n-STAT3 scores were associated with longer RFS. Furthermore, in the two-group comparison of clinical factors and n-STAT3 scores, significant differences were observed in clinical factors such as tumor size, clinical N1b status, and number of LNMs, indicating an association with high-risk factors for recurrence in the low n-STAT3 group. These findings suggest that high n-STAT3 scores are associated with favorable prognosis in patients with PTC. Previous studies of STAT3 in patients with PTC have shown conflicting results as a positive and negative regulator of tumor growth (14); pSTAT3 is significantly upregulated in lymphatic metastases of PTC (8), whereas pSTAT3 negatively regulates PTCs with large tumor size (9, 10) and distant metastases (9).

STAT3 is phosphorylated at Tyr705 by JAK tyrosine kinase upon interaction of various cytokines with their receptors. Phosphorylated STAT3 dimerizes and translocates to the nucleus, where it binds to DNA and induces transcription of target genes (15, 16). Once dephosphorylated, STAT3 is rapidly exported from the nucleus. In this study, nuclear STAT3 expression was evaluated as an indicator of STAT3 activation. The activation of STAT3 is regulated by multiple post-translational modifications. Tyr705 phosphorylation, which is involved in dimer formation, is considered to be the most important modification. Several studies used pSTAT3(Y705) as an indicator of STAT3 activation. However, various regulatory mechanisms, such as phosphorylation of S727, acetylation of lysine residues, methylation, and SUMOylation, have also been reported, which affect STAT3 activity while interfering with each other (12, 14, 17). In the present study, the total amount of STAT3 protein in the nucleus, regardless of post-translational modification, was used as an indicator of STAT3 activation. Non-canonical functions of STAT3, such as mitochondrial regulation, were not evaluated (12, 18).

Cytokines secreted from immune cells surrounding tumor cells can influence STAT3 activation. To investigate the contribution of immune cells to STAT3 activation in PTC, we performed a whole-slide IHC analysis in representative cases from the current cohort. Using a small number of representative cases, we observed no significant differences in the number of tumor-infiltrating lymphocytes (TILs), tertiary lymphoid structures, B cells, T cells, plasma cells, or macrophages between the n-STAT3 high and low groups (Supplementary Figs 7 and 8 and Supplementary Table 1). We did, however, observe a slight tendency toward higher TILs and CD8+ T cells in the n-STAT3-low group. Future experiments should address two possibilities: STAT3 activation is inhibited by TILs and CD8+ T cells, or STAT3 activation is primarily driven by intrinsic tumor-related mechanisms.

Many studies have shown that STAT3 induces the transcription of genes involved in cancer cell proliferation and survival, such as *CCND1*, *BCL2*, and *MYC* (11). However, contrary to these reports, PTC cases with a high n-STAT3 score had a better prognosis, as shown in the present study. This suggests that STAT3 may not induce the expression of these genes in PTC. There are various possible mechanisms by which STAT3 functions as a tumor suppressor. STAT3 may induce genes that suppress cancer progression, such as *FOXO1*, *p14ARF*, and *CDKN1A (P21)*, as previously reported (19). Cout *et al.* reported that STAT3 suppresses tumor growth by inhibiting the Warburg effect in a mouse model of PTC (9). In mammary epithelial cells, STAT3 induces senescence via the TGF beta/SMAD3 pathway and suppresses tumorigenicity (20). Loss of STAT3 and ARF expression in patients with prostate cancer is correlated with an increased risk of disease recurrence and metastasis (21). Further investigation is needed to determine the mechanism by which STAT3 contributes to favorable prognosis in PTC.

*TERT* promoter mutations are known to be associated with a poor prognosis (22, 23). The proportion of cases with a lower n-STAT3 score was higher in the group with *TERT* promoter mutations than in the group without mutations in the present study, supporting the finding that a higher n-STAT3 score is associated with favorable prognosis. In contrast, the proportion of cases with a higher n-STAT3 score was higher in the group of cases with *BRAF* p.V600E mutation than in the group without mutations. Although *BRAF* p.V600E mutation does not have the same impact as *TERT* promoter mutations on prognosis, it is generally recognized as a factor associated with a poor prognosis (24, 25). However, our previous report (4) and that of another group showed that *BRAF* p.V600E mutation alone did not have an impact on prognosis in the Japanese population (26, 27). Race (28), including iodine intake (29), may have been related to this difference.

This study has several limitations. Cases with strong calcification were excluded from the study because tissue decalcification impairs IHC for adequate evaluation. Subjective IHC assessment using the H-scoring system may also have biased the results. The study did not cover all stages of PTC because of the exclusion of cases with nodules ≤10 mm. Furthermore, the IHC investigation was limited to a narrow area of the TMA with a diameter of 1 mm. This suggests that the potential for cancer heterogeneity was not included in this investigation.

In conclusion, n-STAT3 expression is associated with favorable prognosis in cases with PTC. Further studies are needed to investigate how STAT3 contributes to the favorable prognosis of PTCs.

## Supplementary materials



## Declaration of interest

The authors declare that there is no conflict of interest that could be perceived as prejudicing the impartiality of the work reported.

## Funding

This work was supported by JSPS KAKENHI Grant Number JP23K06456.

## Author contribution statement

TC and KT designed the study. IS, AE, KToda, and HM collected clinical data. SB and KM performed immunohistochemistry. CM, TC, and AE reviewed the histopathology and immunohistochemistry results of all the cases and interpreted the results. CM and TC performed the acquisition, analysis, and interpretation of the data and statistical analysis. IS and KTakeuchi equally contributed to drafting and critically revising the manuscript. All authors have reviewed the manuscript.

## Ethics approval and consent to participate

This study was conducted in accordance with the principles of the Declaration of Helsinki (revised in Brazil 2013). Patient consent was obtained for the use of clinical samples for research purposes, according to the regulations defined by the Institutional Review Board of the JFCR (IRB number 2021-GB-102, Mar 11, 2022).

## References

[1] Tuttle RM. Risk-adapted management of thyroid cancer. Endocr Pract 2008 14 764–774. (10.4158/ep.14.6.764) 18996800

[2] Ebina A, Sugitani I, Fujimoto Y, et al. Risk-adapted management of papillary thyroid carcinoma according to our own risk group classification system: is thyroid lobectomy the treatment of choice for low-risk patients? Surgery 2014 156 1579–1588 discussion 1588–1589. (10.23736/s2724-6507.20.03342-8) 25262223

[3] Colombo C, Giancola N & Fugazzola L. Personalized treatment for differentiated thyroid cancer: current data and new perspectives. Minerva Endocrinol 2021 46 62–89. (10.23736/s2724-6507.20.03342-8) 33213119

[4] Ebina A, Togashi Y, Baba S, et al. TERT promoter mutation and extent of thyroidectomy in patients with 1–4 cm intrathyroidal papillary carcinoma. Cancers 2020 12 2115. (10.3390/cancers12082115) 32751594 PMC7464551

[5] Gao S, Zhang W, Yan N, et al. The impact of STAT3 and phospho-STAT3 expression on the prognosis and clinicopathology of ovarian cancer: a systematic review and meta-analysis. J Ovarian Res 2021 14 164. (10.1186/s13048-021-00918-6) 34789292 PMC8600722

[6] Tong M, Wang J, Jiang N, et al. Correlation between p-STAT3 overexpression and prognosis in lung cancer: a systematic review and meta-analysis. PLoS One 2017 12 e0182282. (10.1371/journal.pone.0182282) 28797050 PMC5552221

[7] Ji K, Zhang M, Chu Q, et al. The role of p-STAT3 as a prognostic and clinicopathological marker in colorectal cancer: a systematic review and meta-analysis. PLoS One 2016 11 e0160125. (10.1371/journal.pone.0160125) 27504822 PMC4978497

[8] Zhang J, Gill A, Atmore B, et al. Upregulation of the signal transducers and activators of transcription 3 (STAT3) pathway in lymphatic metastases of papillary thyroid cancer. Int J Clin Exp Pathol 2011 4 356–362. 21577321 PMC3093060

[9] Couto JP, Daly L, Almeida A, et al. STAT3 negatively regulates thyroid tumorigenesis. Proc Natl Acad Sci U S A 2012 109 E2361–E2370. (10.1073/pnas.1201232109) 22891351 PMC3435219

[10] Kim WG, Choi HJ, Kim WB, et al. Basal STAT3 activities are negatively correlated with tumor size in papillary thyroid carcinomas. J Endocrinol Investig 2012 35 413–418. (10.3275/7907) 21897114

[11] Huang S. Regulation of metastases by signal transducer and activator of transcription 3 signaling pathway: clinical implications. Clin Cancer Res 2007 13 1362–1366. (10.1158/1078-0432.ccr-06-2313) 17332277

[12] Huynh J, Chand A, Gough D, et al. Therapeutically exploiting STAT3 activity in cancer – using tissue repair as a road map. Nat Rev Cancer 2019 19 82–96. (10.1038/s41568-018-0090-8) 30578415

[13] Cuello AC, Priestley JV & Sofroniew MV. Immunocytochemistry and neurobiology. Q J Exp Physiol 1983 68 545–578. (10.1113/expphysiol.1983.sp002748) 6139841

[14] Sosonkina N, Starenki D & Park JI. The role of STAT3 in thyroid cancer. Cancers 2014 6 526–544. (10.3390/cancers6010526) 24662939 PMC3980610

[15] Johnson DE, O'Keefe RA & Grandis JR. Targeting the IL-6/JAK/STAT3 signalling axis in cancer. Nat Rev Clin Oncol 2018 15 234–248. (10.1038/nrclinonc.2018.8) 29405201 PMC5858971

[16] Al Zaid Siddiquee K & Turkson J. STAT3 as a target for inducing apoptosis in solid and hematological tumors. Cell Res 2008 18 254–267. (10.1038/cr.2008.18) 18227858 PMC2610254

[17] Verhoeven Y, Tilborghs S, Jacobs J, et al. The potential and controversy of targeting STAT family members in cancer. Semin Cancer Biol 2020 60 41–56. (10.1016/j.semcancer.2019.10.002) 31605750

[18] Darnell JE Jr. STAT3, HIF-1, glucose addiction and Warburg effect. Aging 2010 2 890–891. (10.18632/aging.100239) 21149895 PMC3034174

[19] Carpenter RL & Lo HW. STAT3 target genes relevant to human cancers. Cancers 2014 6 897–925. (10.3390/cancers6020897) 24743777 PMC4074809

[20] Bryson BL, Junk DJ, Cipriano R, et al. STAT3-mediated SMAD3 activation underlies oncostatin M-induced senescence. Cell Cycle 2017 16 319–334. (10.1080/15384101.2016.1259037) 27892764 PMC5324753

[21] Pencik J, Schlederer M, Gruber W, et al. STAT3 regulated ARF expression suppresses prostate cancer metastasis. Nat Commun 2015 6 7736. (10.1038/ncomms8736) 26198641 PMC4525303

[22] Meng Z, Matsuse M, Saenko V, et al. TERT promoter mutation in primary papillary thyroid carcinoma lesions predicts absent or lower (131) i uptake in metastases. IUBMB Life 2019 71 1030–1040. (10.1002/iub.2056) 31026111

[23] Yang X, Li J, Li X, et al. TERT promoter mutation predicts radioiodine-refractory character in distant metastatic differentiated thyroid cancer. J Nucl Med 2017 58 258–265. (10.2967/jnumed.116.180240) 27493271

[24] Chen Y, Sadow PM, Suh H, et al. BRAF(V600E) is correlated with recurrence of papillary thyroid microcarcinoma: a systematic review, multi-institutional primary data analysis, and meta-analysis. Thyroid 2016 26 248–255. (10.1089/thy.2015.0391) 26671072

[25] Attia AS, Hussein M, Issa PP, et al. Association of BRAF(V600E) mutation with the aggressive behavior of papillary thyroid microcarcinoma: a meta-analysis of 33 studies. Int J Mol Sci 2022 23 15626. (10.3390/ijms232415626) 36555268 PMC9779545

[26] Ito Y, Yoshida H, Maruo R, et al. BRAF mutation in papillary thyroid carcinoma in a Japanese population: its lack of correlation with high-risk clinicopathological features and disease-free survival of patients. Endocr J 2009 56 89–97. (10.1507/endocrj.k08e-208) 18840924

[27] Matsuse M, Yabuta T, Saenko V, et al. TERT promoter mutations and Ki-67 labeling index as a prognostic marker of papillary thyroid carcinomas: combination of two independent factors. Sci Rep 2017 7 41752. (10.1038/srep41752) 28150740 PMC5288691

[28] Rashid FA, Munkhdelger J, Fukuoka J, et al. Prevalence of BRAF(V600E) mutation in Asian series of papillary thyroid carcinoma-a contemporary systematic review. Gland Surg 2020 9 1878–1900. (10.21037/gs-20-430) 33224863 PMC7667088

[29] Kim HJ, Park HK, Byun DW, et al. Iodine intake as a risk factor for BRAF mutations in papillary thyroid cancer patients from an iodine-replete area. Eur J Nutr 2018 57 809–815. (10.1007/s00394-016-1370-2) 28258306

