# Supplementary material for: Nuclear STAT3 expression is associated with favorable prognosis in papillary thyroid carcinoma
Source: Eur Thyroid J. 2025 Nov 3;14(6):e250080. doi: 10.1530/ETJ-25-0080 (PMC12586974; doi:10.1530/ETJ-25-0080)
Supplement: Supplementary file 1 [file supplementary_materials.pdf]

Supplementary Table 1. Correlations between n-STAT3 and immune cell profiles based on whole-slide evaluation, focusing on tumor-infiltrating lymphocytes (TILs), tertiary lymphoid structures (TLS), and the presence of Hashimoto’s thyroiditis.

	n-STAT3	
	High (n=6)	Low (n=6)
TILs (≧10%)	2	3
TLS	3	3
Hashimoto	1	2

*TILs: tumor-infiltrating lymphocytes, TLS: tertiary lymphoid structures.

Supplementary Figures

Supplementary Figure 1.

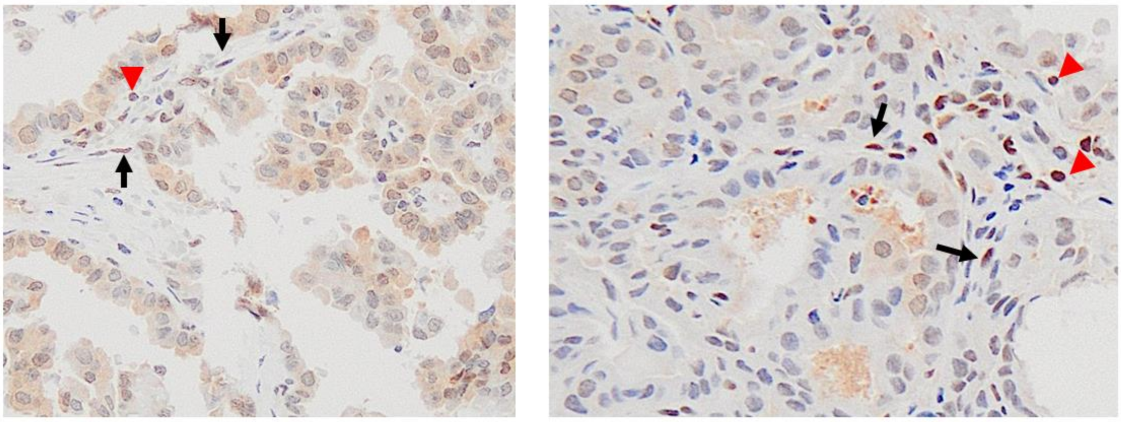

Supplementary Figure 1. STAT3 scoring and exclusion.

STAT3 immunoreactivity in the nuclei of papillary thyroid carcinoma (PTC) cells was evaluated. The nuclei of PTC cells are characteristically enlarged and irregularly shaped, allowing for reliable morphological identification. Vascular endothelial cells (indicated by black arrows), which exhibit spindled nuclei, and immune cells (indicated by red arrowheads), which have small round nuclei were morphologically excluded from the H-score evaluation.

Supplementary Figure 2.

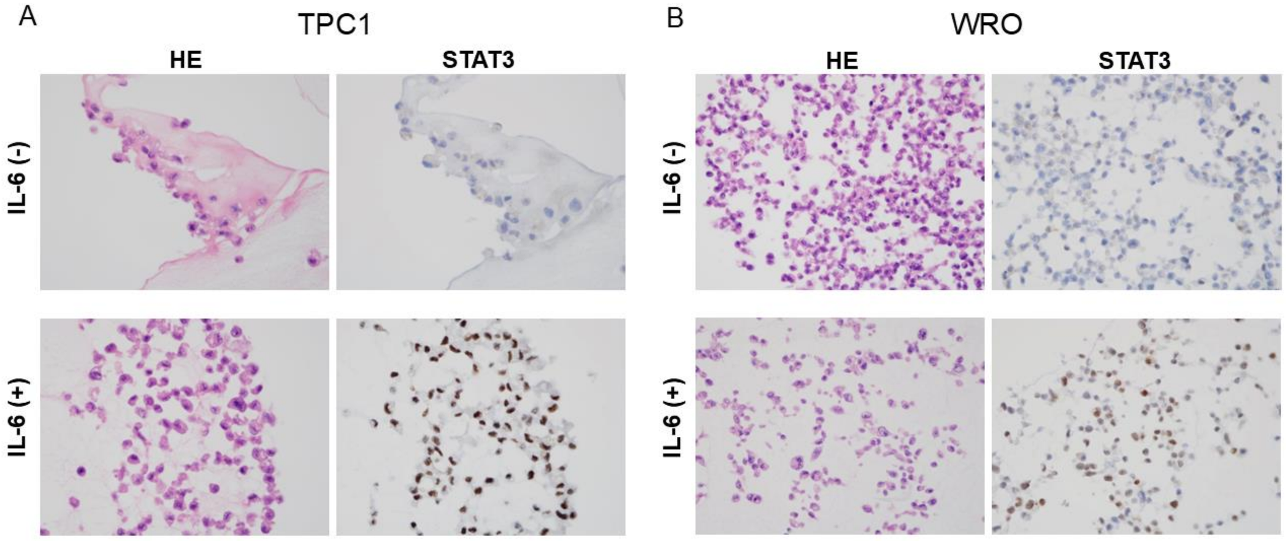

Supplementary Figure 2. STAT3 staining of papillary thyroid carcinoma cell lines with or without IL-6 stimulation.

Two papillary thyroid carcinoma (PTC) cell lines, TPC1 (A) and WRO (B), were cultured in DMEM supplemented with 10% fetal bovine serum and antibiotics, and stimulated with a combination of IL-6 (50ng/ml) and soluble IL-6 receptor α (100ng/ml) for 15 min. Cell blocks were prepared and subjected to STAT3 immunohistochemistry. In both cell lines, IL-6 stimulation resulted in a marked induction of n-STAT3, whereas only minimal n-STAT3 immunoreactivity was observed in unstimulated control cells.

Supplementary Figure 3A.

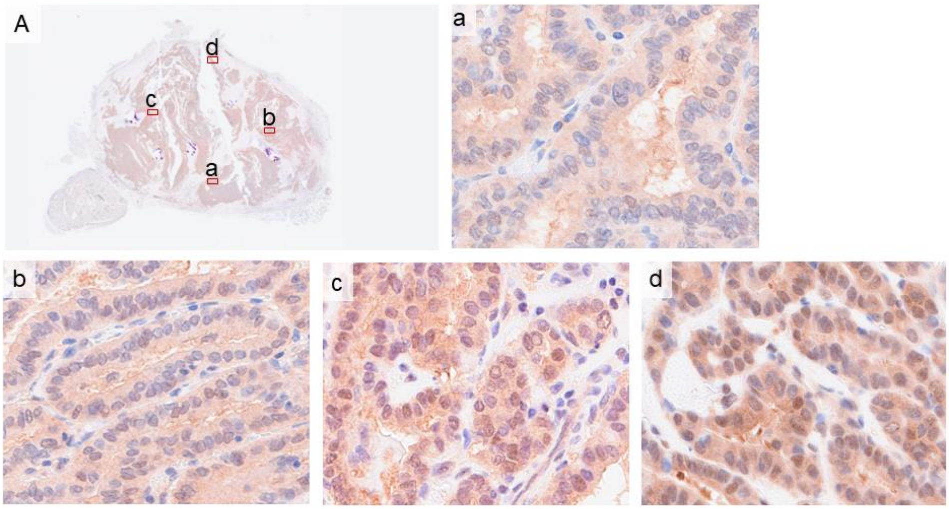

Supplementary Figure 3B.

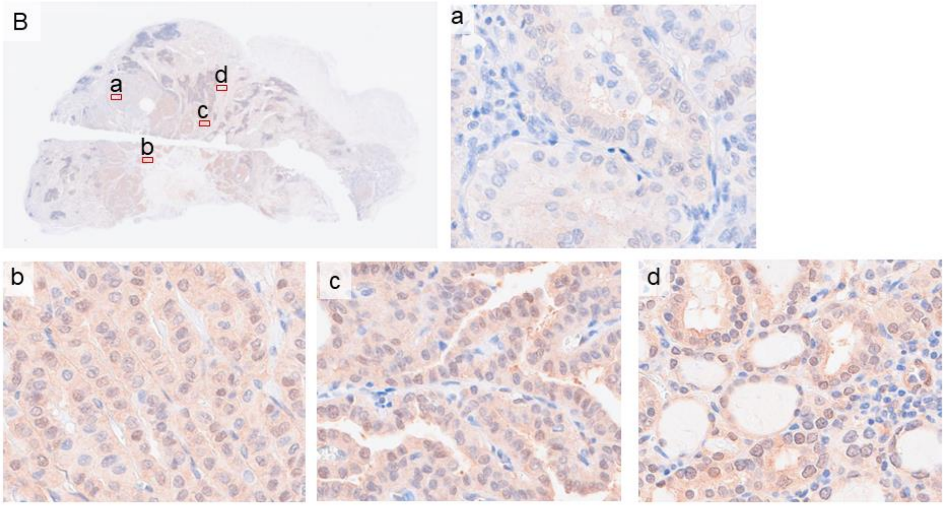

Supplementary Figure 3C.

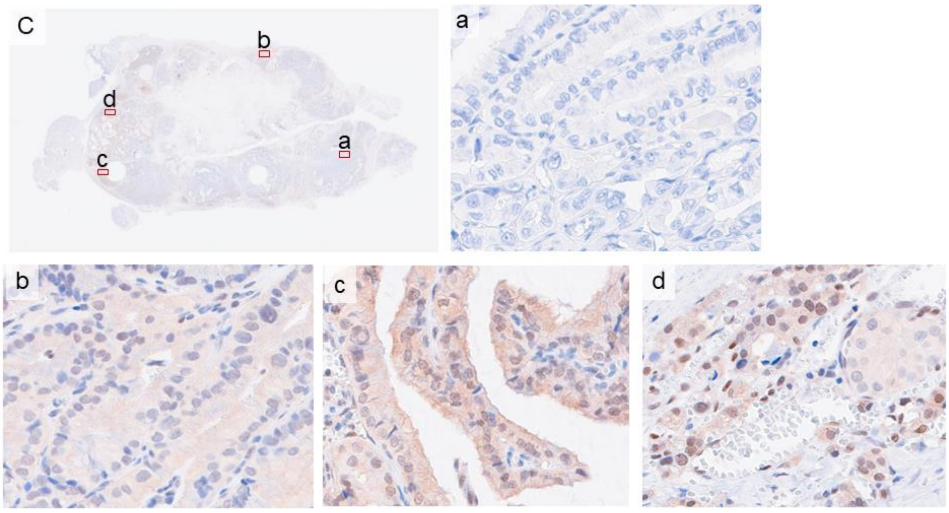

Supplementary Figure 3D.

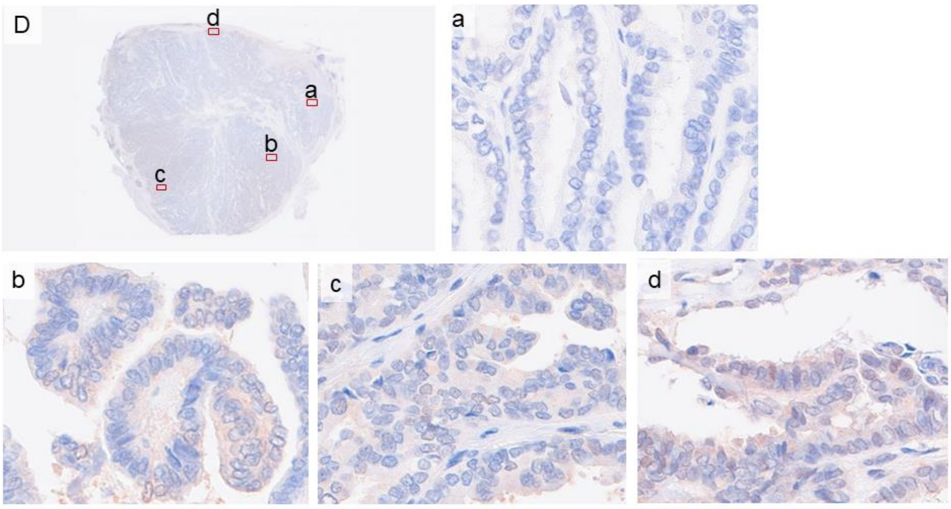

Supplementary Figure 3. Verification of intratumoral heterogeneity in STAT3 staining using whole-slide sections.

STAT3 immunostaining was performed on whole-slide sections from 12 patients with PTC. Representative images from four different areas in four selected cases are shown. H-scores for n-STAT3 were calculated based on whole-slide sections and compared with the corresponding scores obtained from tissue microarrays (TMAs). In the TMA analysis, Cases A-C exhibited high H-scores (≥70), whereas Case D showed a low

score (<70).

(A) Case A: TMA H-score = 190. Whole-slide regions: a = 40, b = 110, c = 160, d = 160.

(B) Case B: TMA H-score = 100. Whole-slide regions: a = 20, b = 110, c = 110, d = 140.

(C) Case C: TMA H-score = 120. Whole-slide regions: a = 0, b = 80, c = 120, d = 140.

(D) Case D: TMA H-score = 40. Whole-slide regions: a = 20, b = 20, c = 90, d = 120.

Supplementary Figure 4.

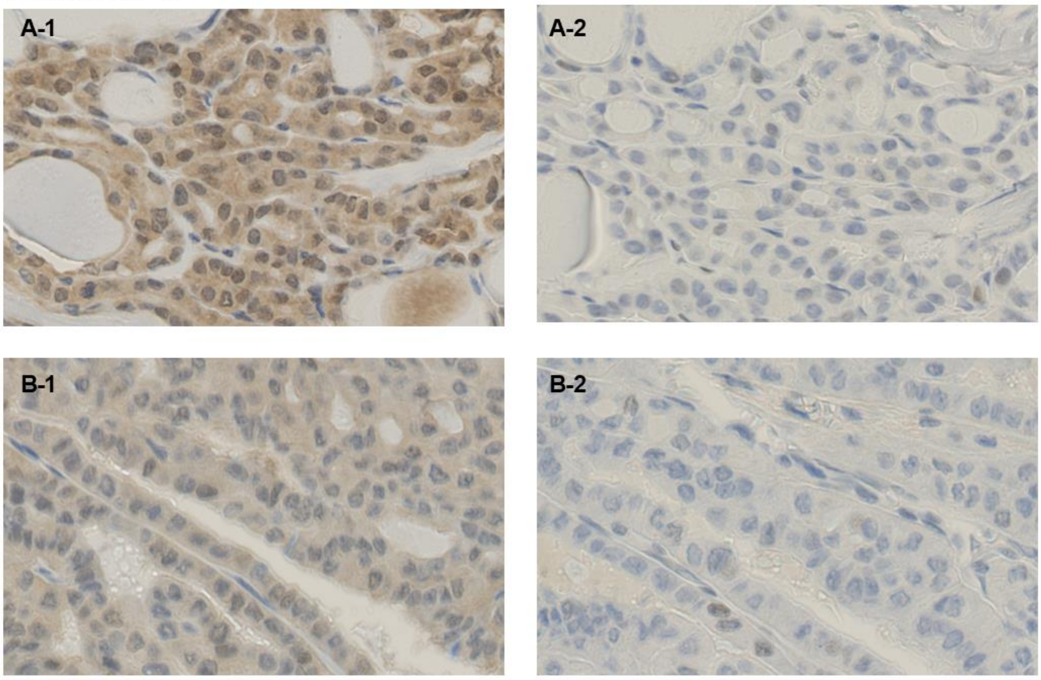

Supplementary Figure 4. Representative images of immunohistochemistry using STAT3(124H6) and pSTAT3(Y705).

A. Case 1.

B. Case 2.

The staining intensity in the nucleus is weaker for pSTAT3 (A-2, B-2) than for STAT3 (A-1, B-1).

Supplementary Figure 5.

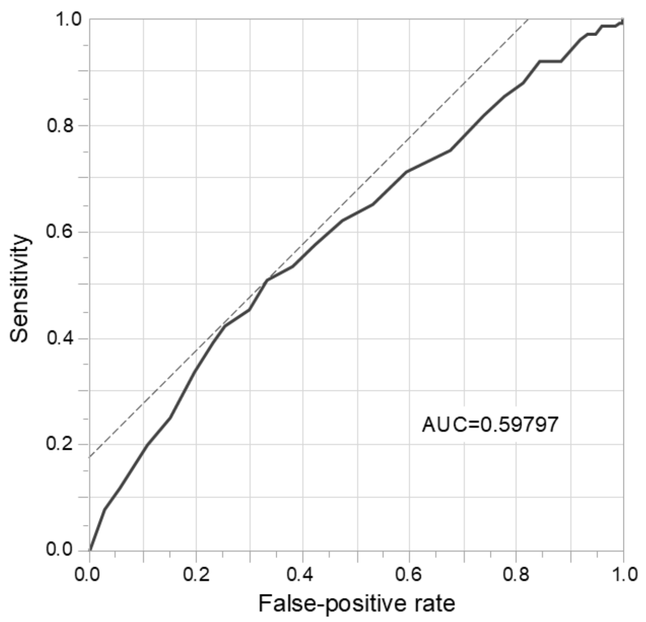

Supplementary Figure 5. Receiver-operating characteristic (ROC) curve analysis

The cutoff point calculated from the ROC curve based on the presence or absence of recurrence is 70 (AUC=0.598).

Supplementary Figure 6.

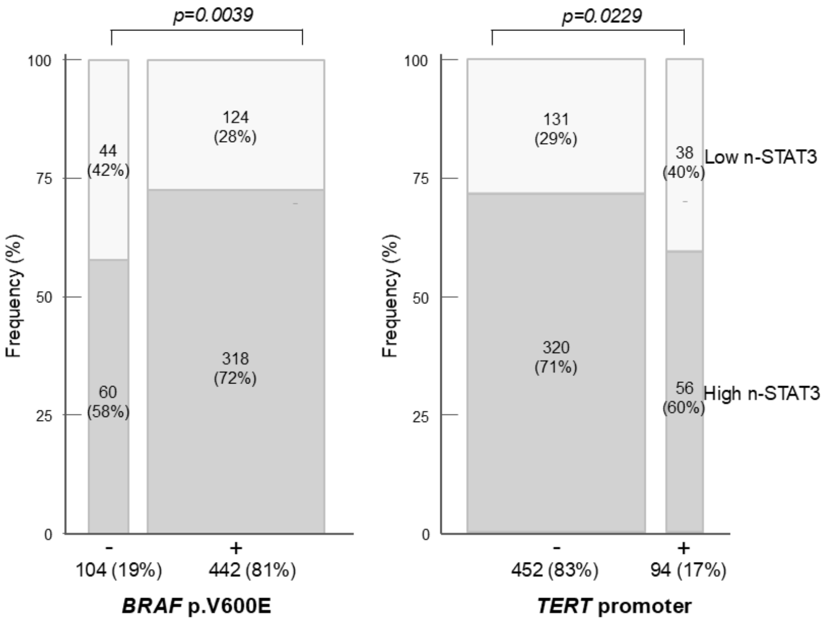

Supplementary Figure 6. Correlations of *BRAF* p.V600E and *TERT* promoter mutations with n-STAT3 expression

BRAF p.V600E and *TERT* promoter mutations were detected in 546 (48%) patients. Techniques for these mutations have been reported previously [4]. *BRAF* p.V600E mutation is positive in 81% of cases, and the percentage of high n-STAT3 cases is significantly higher in *BRAF* p.V600E-positive cases (72%) than in negative cases (58%) (p=0.0039). High n-STAT3 levels are significantly less frequent in *TERT* promoter-positive cases (60%) than in negative cases (71%) (p=0.0229).

Supplementary Figure 7.

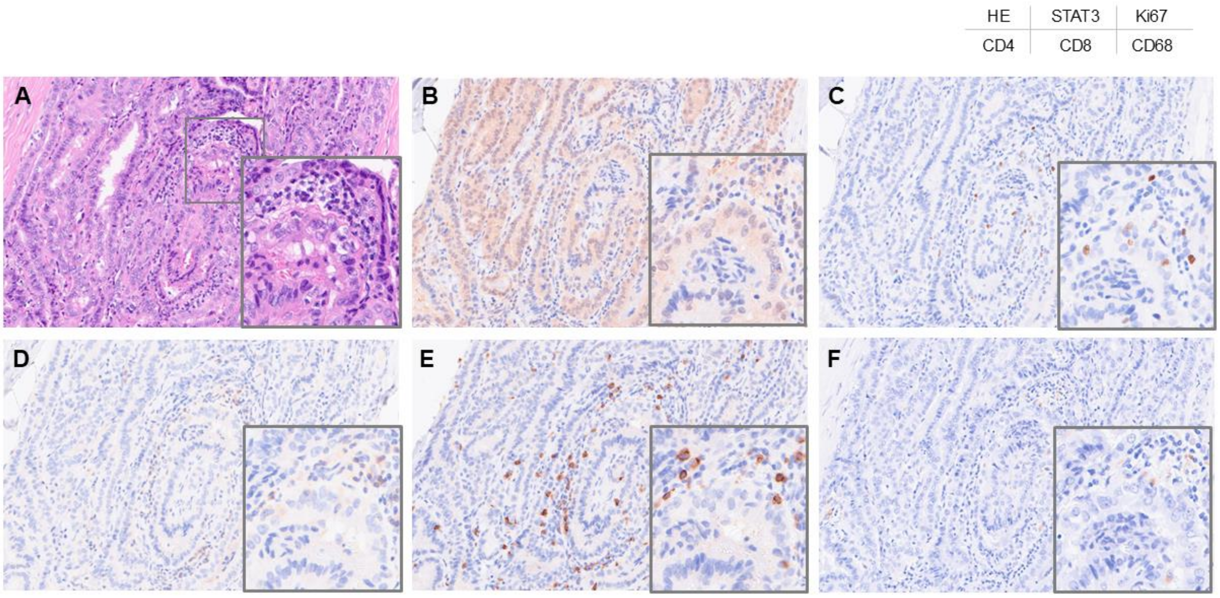

Supplementary Figure 7. Representative images of immune cells in papillary thyroid carcinomas, visualized by immunohistochemistry on whole-slide consecutive sections.

Panels: A: H&E, B: STAT3, C: Ki67, D: CD4, E: CD8, F: CD68. Insets show magnified views of the region indicated in panel A. IHC on consecutive sections showed no spatial concordance between immune cell localization and n-STAT3 expression in PTC cells.

Supplementary Figure 8.

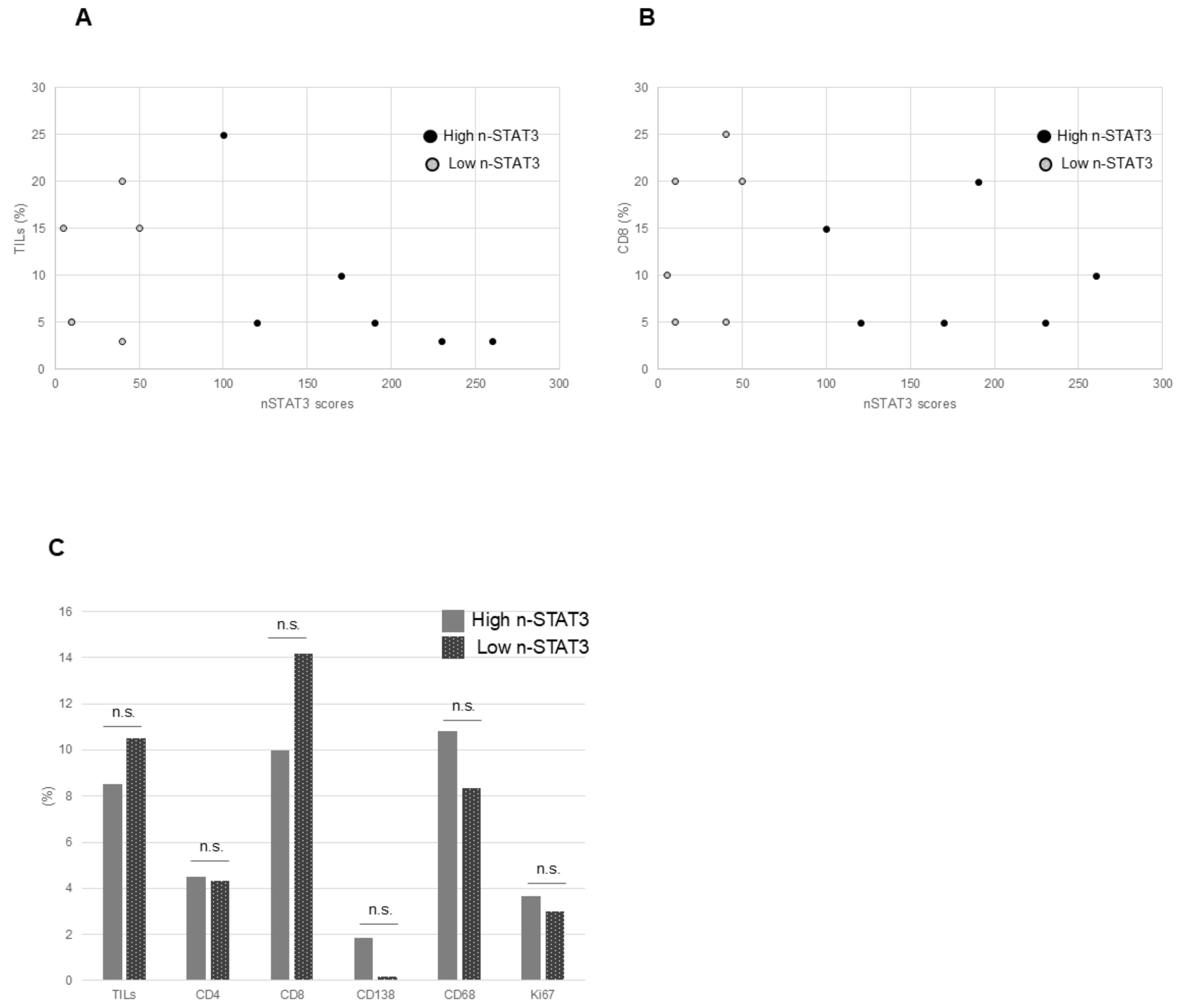

Supplementary Figure 8. Evaluation of immune cells in papillary thyroid carcinoma using whole-slide sections.

A: Scatter plots showing n-STAT3 scores and tumor-infiltrating lymphocytes (TILs) in whole-slide sections from 12 representative cases.

B: Scatter plots showing n-STAT3 scores and CD8-positive T cell infiltrations, assessed in tumor regions using the same method as for TIL evaluation, based on whole-slide sections from 12 representative cases.

C: Comparison of median counts for inflammatory cell marker-positive cells between n-STAT3 high and low groups (6 cases each), based on whole-slide evaluation. *n.s.: not significant.
